# Comparative effectiveness of low versus high dose upadacitinib as maintenance treatment in patients with ulcerative colitis: a real-world cohort study from the United States

**DOI:** 10.1093/crocol/otag028

**Published:** 2026-05-08

**Authors:** Priya Sehgal, Gursimran S Kochhar, Harpreet Kaur, Himsikhar Khataniar, Raina Shivashankar, James D Lewis, Aakash Desai

**Affiliations:** Division of Gastroenterology and Hepatology, Thomas Jefferson University Hospital, Philadelphia, PA, United States; Division of Gastroenterology, Hepatology, and Nutrition, Allegheny Health Network, Pittsburgh, PA, United States; Division of Gastroenterology, Hepatology, and Nutrition, Allegheny Health Network, Pittsburgh, PA, United States; Department of Medicine, Allegheny Health Network, Pittsburgh, PA, United States; Division of Gastroenterology and Hepatology, Thomas Jefferson University Hospital, Philadelphia, PA, United States; Division of Gastroenterology and Hepatology, Perelman School of Medicine, University of Pennsylvania, Philadelphia, PA, United States; Division of Gastroenterology, Hepatology, and Nutrition, Allegheny Health Network, Pittsburgh, PA, United States

**Keywords:** upadacitinib, ulcerative colitis, maintenance dose

## Abstract

**Background:**

There is limited real-word data comparing efficacy of low dose upadacitinib (UPA) to high dose UPA as maintenance therapy for ulcerative colitis (UC).

**Methods:**

This was a retrospective cohort study utilizing the U.S. Collaborative Network in adults ≥18 years old with UC who initiated UPA 15 mg compared to 30 mg for maintenance therapy between April 2022 and December 2023. The primary outcome was a composite of intravenous steroid use, oral steroid use, or colectomy from 12 to 60 weeks from the index UPA prescription. Propensity score matching (PSM) was performed for demographics, co-morbid conditions, laboratory, and IBD medication history. Cox proportional hazard model was used to identify predictors of failure.

**Results:**

Among 1110 patients on UPA maintenance therapy, 361 (32.5%) were on 15 mg and 749 (67.5%) were on 30 mg. After PSM, there was no difference in the composite outcome of steroid use or colectomy between the 15-mg UPA cohort versus 30 mg cohort (35.3% vs 35.6%; aHR 0.95, 0.71-1.24, *P* = .8). There was no difference in IV steroid use, oral steroid use or change in therapy between the two cohorts. There was no difference in the proportion of patients who achieved a fecal calprotectin of <250 μg/g (55.8% vs 63.5%, *P* = .33). Recent oral or IV steroid use and rheumatoid arthritis were associated with failure of both 15 mg and 30 mg UPA.

**Conclusion:**

Our study indicates that 15 mg UPA shows similar efficacy as 30 mg UPA for maintenance treatment in a subset of patients with UC.

## Introduction

Ulcerative colitis (UC) is a chronic, relapsing inflammatory disease which results in mucosal inflammation that typically extends from the rectum to the proximal colon.^1^^,^^2^ In recent years, the therapeutic options for moderate to severely active UC have expanded significantly, partly driven by the introduction of small molecule therapies. These therapies offer several advantages including oral administration, rapid onset of action and no risk of immunogenicity compared to biologic agents.^3^^,^^4^

Upadacitinib is an oral, selective and reversible Janus kinase (JAK) inhibitor approved for the treatment of moderate to severely active UC.^2^^,^^5^^,^^6^ In the U-ACHIEVE and U-ACCOMPLISH induction trials, 987 patients were treated with upadacitinib 45 mg once daily, of whom 681 achieved a clinical response and were subsequently enrolled in the U-ACHIEVE maintenance study.^2^ In the maintenance phase, both upadacitinib 15 mg and 30 mg once daily significantly outperformed placebo in achieving the primary endpoint of clinical remission at week 52 (40.4% and 53.6% vs 10.8%, respectively both <0.001).^2^ Although both maintenance doses were effective, the higher 30 mg dose appeared numerically more effective.^2^ In a subsequent analysis of the U-ACHIEVE data, it was noted that by week 52, 20% more patients in the upadacitinib 30 mg groups were in a less severe disease state than patients in the upadacitinib 15 mg group as per the adapted Mayo score. (*P* < .0001).^7^ Patients enrolled in clinical trials do not always reflect those who receive a medication in routine clinical care. There are no real world studies available comparing upadacitinib 15 mg versus 30 mg for maintenance therapy in UC. The approved prescribing instructions recommend using the lowest effective dosage needed to maintain response.^8^

The present study compared the effectiveness of upadacitinib 15 mg versus upadacitinib 30 mg for the maintenance treatment of ulcerative colitis using a real-world database. The primary aim of this study was to assess the risk of a composite outcome including hospitalization requiring intravenous steroids, oral steroid use and/or colectomy between the high dose and low dose maintenance upadacitinib cohort. The hypothesis was that the high dose cohort would be associated with a reduced risk of the composite outcome when compared to the low dose cohort.

## Materials and methods

### Database

A retrospective cohort study was conducted using the U.S. Collaborative Network in TriNetX (Cambridge, MA, USA), a multi-institutional database. TriNetX is a global federated research network which provides real-time access to de-identified electronic health records of more than 114 million patients within 69 health care organizations in the United States. Most health care organizations are large academic medical institutions which contain inpatient and outpatient facilities. The data represents the entire patient population of the organization. The de-identification process is determined and done at a network-level and attested through a formal determination by a qualified expert as defined in the HIPAA Privacy Rule. TriNetX obfuscates patient counts <10 to ensure patient anonymity. Clinical variables are derived directly from electronic health records of included health care organizations as well as retrieved through a built-in natural language processing system that extracts variables from clinical documents. Robust quality assurance is achieved at the time of extraction from electronic health records before inclusion in the database, in a systematic and standardized format. The process also includes data cleaning which rejects patient records that don’t meet the TriNetX quality standards. The database does not include claims data or data collected from randomized clinical trials. The database includes patient data regarding demographics, diagnosis, procedures, laboratory values, and medications. The interface only provides aggregate counts and statistical summaries to protect patient health information and ensures that the data remain de-identified at all levels of data retrieval and dissemination.

### Study participants and cohorts

We conducted a real-time search and analysis of the US Collaborative Network in the TriNetX platform. Patients who were diagnosed with UC on upadacitinib included adults age ≥ 18 years old who had an International Classification of Disease, Tenth Revision, Clinical Modification (ICD-10-CM) codes in their EHR for Ulcerative colitis (K51*) and a RxNorm code (2196092) for upadacitinib between April 1, 2022, and December 31, 2023. Patients who initiated maintenance dosing of upadacitinib were divided into those who were on 15 mg (low dose cohort) and 30 mg (high dose cohort). Patients who had received a prescription for an alternative dose prior to the prescription of low or high dose upadacitinib and within 8 weeks of index upadacitinib prescription were excluded. For example, in the high dose cohort, patients were excluded if they had a 15-mg upadacitinib prescription prior to a 30-mg prescription and within 8 weeks of the index upadacitinib prescription. Of note, there were no patients who received prescription of 15 mg upadacitinib after the 30-mg prescription. This was done to avoid potential overlap of patients in the low and high dose cohorts. Additionally, patients who underwent colectomy or were switched to an alternative therapy within 12 weeks of index upadacitinib prescription were also excluded. This would ensure patients who were initiated on maintenance therapy had sufficient trial of the maintenance upadacitinib dose. Furthermore, patients who received a prescription for steroids within 30 days and 12 weeks of upadacitinib prescription, in either cohort, were still included and this was noted in the final analysis.

### Study outcomes

The primary outcome of the study was to assess the risk of a composite outcome of hospitalization requiring intravenous steroids, oral steroid use and/or colectomy between 12 and 60 weeks from the index upadacitinib prescription between low dose and high dose cohort. Outcome ascertainment was performed after 12 weeks instead of 8 weeks to account for potential delays in initiation of therapy and insurance approval. Patients with hospitalization requiring intravenous steroids were identified using RxNorm code for methylprednisolone or hydrocortisone. Patients with oral steroid use were identified using RxNorm code for prednisone. Patients who required colectomy were identified using the Current Procedural Terminology (CPT) codes. The secondary outcomes were to assess the risk of intravenous steroid use, oral steroid use, colectomy, and change in therapy. We also assessed the proportion of patients who had fecal calprotectin < 250 micrograms/gram and C-reactive protein < 8 gram/L (g/L) between the two cohorts in patients who had available data. A sensitivity analysis was conducted to assess all outcomes after 8 weeks from the index UPA prescription.

The secondary aim of the study was to identify individual predictors of failure, defined by the composite outcome, for the low dose and high dose cohorts. Co-variates were assessed within the preceding 1 year of upadacitinib prescription, and the most recent available values were obtained. Co-variates included age, male sex, CRP ≥ 12 mg/L, albumin < 2.5 mg/dL, hemoglobin < 10 g/dL, nicotine dependence, primary sclerosing cholangitis, obesity, Clostridioides difficile infection, rheumatoid arthritis, psoriasis, prednisone use, methylprednisolone use, previous exposure to vedolizumab and/or ustekinumab.

### Statistical analysis

All statistical analyses were conducted using the TriNetX software using the browser- based real-time analytics feature, TriNetx Live (TriNetX LLC, Cambridge, MA). Baseline characteristics of cohorts were described using means, standard deviations, and proportions. Covariates based on demographics, comorbid diseases, laboratory parameters, and historical IBD medication use were identified. One-to-one (1:1) propensity score matching was performed to balance the following covariates between groups: advanced therapy and intravenous and/or oral steroid use in the preceding 1 year, age, sex, race, obesity, diabetes mellitus, nicotine dependence (defined by ICD-10 code), primary sclerosing cholangitis, rheumatoid arthritis, ankylosing spondylitis, psoriasis, mean hemoglobin, albumin, C-reactive protein, and fecal calprotectin. TriNetX platform utilizes input matrices of the user-identified covariates to conduct logistic regression analysist to obtain propensity scores for all individual subjects. The propensity scores generated are used to match patients using greedy nearest-neighbor algorithms with a caliper width of 0.1 pooled standard deviations. TriNetX randomizes the order of rows to eliminate bias resulting from nearest-neighbor algorithms. Standardized mean difference after propensity score matching indicate the success of matching a covariate between the two cohorts. A standardized mean difference < 0.1 indicates that the difference between the cohorts for the co-variate is small. After propensity score matching, the risk of each outcome was calculated and expressed as adjusted hazard ratios (aHR) with 95% confidence intervals (CIs). Cox proportional hazard model was used to estimate the impact of different covariates on the risk of the primary outcome between the low dose and high dose cohort. Co-variates included in the model were age, sex, nicotine dependence, C-reactive protein ≥ 12 g/L, albumin < 2.5 g/dL, moderate-severe anemia (hemoglobin < 10 g/dL), corticosteroid use, obesity, Clostridioides difficile infection, ustekinumab use, vedolizumab use, and other autoimmune diseases. T tests were utilized for non-time-to-event outcomes.

### Ethical considerations

Not applicable in setting of database study.

## Results

### Characteristics of study population

We identified 1110 patients with UC who were on maintenance therapy of upadacitinib, of which 361 (32.5%) were on 15 mg (low dose cohort) and 749 (67.5%) were on 30 mg (high dose cohort). Before PSM, the low dose cohort was older (46.3 ± 17.2 vs 39.6 ± 14.9, *P* < .0001), had a lower proportion of male sex (39.6% vs 50.2%, *P* = .0009) and higher proportion of White race (80% vs 73.5%, *P* = .01). There were higher proportion of patients with concomitant autoimmune diseases in the low dose compared to the high dose cohort. There was no difference in the proportion of patients who received oral steroids or intravenous steroids in the preceding one year between the two cohorts (Table 1). Before PSM, there were lower proportion of patients who were exposed to 2 or more advanced therapies in the low dose cohort compared to high dose cohort (39.3% vs 56.3%, *P* < .0001). After PSM, the co-variates were well balanced between the two cohorts (Table 1). Additionally, there was no difference in the proportion of patients who received prednisone within 30 days (22.1% vs 23.3%, *P* = .65) and 12 weeks (28.9% vs 28.1%, *P* = .78) between the two cohorts following the index upadacitinib prescription. There was no difference in the proportion of patients with history of tofacitinib prescription between the two cohorts (18.8% for 15 mg dosing vs 16.2% for 30 mg dosing, *P* = .28).

**Table 1:** Baseline characteristics of patients in the low dose and high dose cohort before and after propensity score matching.

	Before propensity score matching			After propensity score matching		
	Low dose cohort (*n* = 361)	High dose cohort (*n* = 749)	SMD	Low dose cohort (*n* = 283)	High dose cohort (*n* = 283)	SMD
**Demographics**						
** Age at index (mean ± SD)**	46.3 ± 17.2	39.6 ± 14.9	0.41	42.8 ± 16.2	43.2 ± 15.6	0.02
** Male sex**	143 (39.6%)	376 (50.2%)	0.21	135 (46.2%)	126 (43.1%)	0.06
** Race**						
** White**	289 (80%)	551 (73.5%)	0.15	231 (79.1%)	231 (79.1%)	0.0001
** African American**	21 (5.8%)	51 (6.8%)	0.04	17 (5.8%)	11 (3.7%)	0.09
** Hispanic**	18 (4.9%)	46 (6.1%)	0.05	15 (5.1%)	12 (4.1%)	0.04
**Medications** ^a^						
** Prednisone**	215 (59.5%)	459 (61.2%)	0.03	173 (59.2%)	184 (63%)	0.07
** Methylprednisolone**	125 (34.6%)	214 (28.5%)	0.13	89 (30.4%)	98 (33.5%)	0.06
** Budesonide**	91 (25.2%)	263 (35.1%)	0.21	81 (27.7%)	87 (29.7%)	0.04
** TNFi**	128 (35.4%)	209 (27.9%)	0.16	90 (30.8%)	100 (34.2%)	0.07
** Vedolizumab**	31 (8.5%)	105 (14%)	0.17	30 (10.2%)	34 (11.6%)	0.04
** Ustekinumab**	63 (17.4%)	194 (25.9%)	0.20	60 (20.5%)	64 (21.9%)	0.03
** Azathioprine**	20 (5.5%)	59 (7.8%)	0.09	18 (6.1%)	18 (6.1%)	0.0001
** Mercaptopurine**	10 (2.7%)	23 (3%)	0.01	10 (3.4%)	10 (3.4%)	0.0001
**Labs** ^a^						
** Hemoglobin (g/dL)**	13.1 ± 1.7	12.9 ± 1.9	0.08	13.2 ± 1.7	12.8 ± 1.8	0.21
**Albumin (g/dL)**	4.01 ± 0.4	4 ± 0.5	0.03	4.02 ± 0.4	3.9 ± 0.5	0.15
** C-reactive protein (mg/L)**	14.5 ± 25.5	15 ± 28.2	0.0.1	13.5 ± 22.2	14.9 ± 28.2	0.05
** Calprotectin (μg/g)**	1131 ± 1210	1118 ± 1151	0.01	1143 ± 1220	1113 ± 1017	0.02
**Co-morbid conditions**						
** Diabetes mellitus**	33 (9.1%)	41 (5.4%)	0.14	17 (5.8%)	24 (8.2%)	0.09
** Nicotine dependence**	20 (5.5%)	20 (2.6%)	0.14	10 (3.4%)	14 (4.7%)	0.06
** Primary sclerosing cholangitis**	10 (2.7%)	31 (4.1%)	0.07	10 (3.4%)	10 (3.4%)	0.0001
** Obesity**	91 (25.2%)	127 (16.9%)	0.20	57 (19.5%)	59 (20.2%)	0.01
** Rheumatoid arthritis**	63 (17.4%)	13 (1.7%)	0.55	10 (3.5%)	12 (4.2%)	0.03
** Ankylosing spondylitis**	18 (4.9%)	10 (1.3%)	0.20	10 (3.5%)	10 (3.5%)	0.0001
** Psoriasis**	38 (10.5%)	24 (3.2%)	0.29	20 (7.0%)	20 (7.0%)	0.0001

Abbreviations: SMD, standardized mean difference; SD, standard deviation; TNFi, tumor necrosis factor inhibitor.

a Laboratory values and medications within 1 year prior to the initiation of upadacitinib.

### Outcomes of low dose versus high dose maintenance upadacitinib

**Figure 1:**
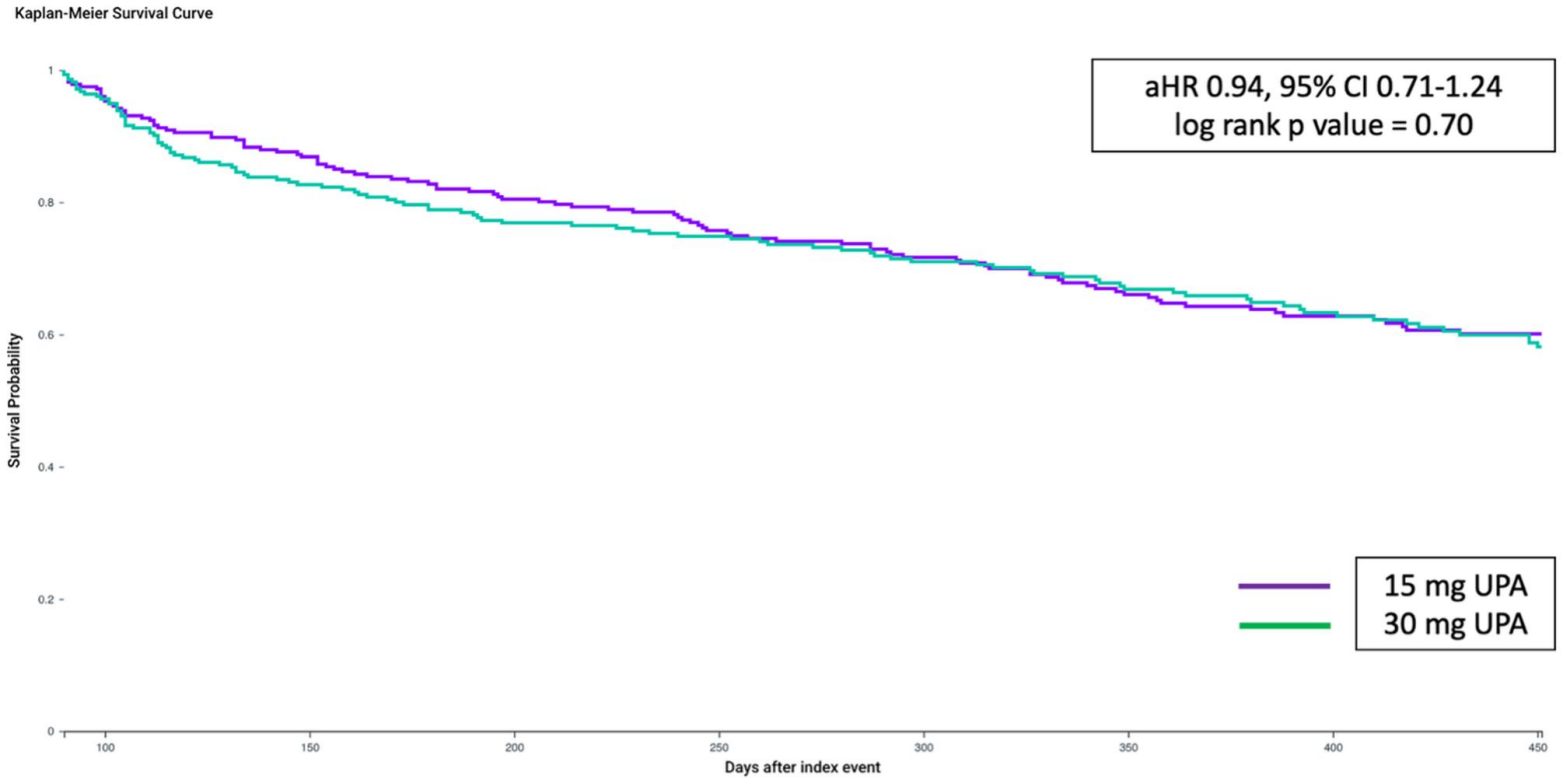
Kaplan-Meier curve for composite outcome of steroid-free and/or colectomy-free survival between the low dose and high dose cohort of patients with ulcerative colitis on upadacitinib.

There were 100 patients in the low dose cohort and 101 patients in the high dose cohort that met the primary outcome. After PSM, there was no difference in the risk of the composite outcome of intravenous steroids, oral steroids or colectomy between the low dose and high dose cohort (35.3% vs 35.6%, aHR 0.95, 95% CI, 0.71-1.24) (Figure 1). There was also no difference in the risk of IV steroid use (17.3% vs 15.5%, aHR 1.07, 95% CI, 0.71-1.61), oral steroid use (27.5% vs 25.4%, aHR 1.06, 95% CI, 0.77-1.46) and change in therapy (15.1% vs 15.1%, aHR 0.95, 95% CI, 0.62-1.45) (Table 2). Fewer than 10 patients required colectomy in both the cohorts. There was also no difference in the proportion of patients with FCP < 250 μg/g in the low dose compared to high dose cohort (55.8% vs 63.5%, *P* = .33). There was no difference in the risk of the outcomes after sensitivity analysis based on outcome ascertainment after 8 weeks between the two cohorts (Table 3).

There was no difference in the risk of the composite outcome in a sub-group analysis in patients who were exposed to 2 or more advanced therapies in the low dose compared to the high dose cohort (42.4% vs 40.9%, aHR 1.03, 95% CI, 0.71-1.50). Additionally, there was no difference in the risk of IV steroid use, oral steroid and change in therapy between the two cohorts (Table 4).

**Table 2:** Comparison of outcomes in patients with UC on Upadacitinib low dose (15 mg) versus high dose (30 mg) for maintenance treatment after propensity score matching.

Outcomes	Cohorts	*N* (%)	aHR	95% CI	*P* value
**Composite outcome**	Low dose	100 (35.3)	0.94	0.71-1.24	.70
	High dose	101 (35.6)			
**IV steroid use**	Low dose	49 (17.3)	1.07	0.71-1.61	.72
	High dose	44 (15.5)			
**Colectomy**	Low dose	<10	—	—	—
	High dose	<10			
**Oral steroid use**	Low dose	78 (27.5)	1.06	0.77-1.46	.70
	High dose	72 (25.4)			
**Change in therapy**	Low dose	43 (15.1)	0.95	0.62-1.45	.83
	High dose	43 (15.1)			
**FCP <250 μg/g** ^a^	Low dose	38 (55.8)	—	—	.33
	High dose	54 (63.5)			
**C reactive protein <8 mg/L**	Low dose	53 (49)	—	—	.19
	High dose	58 (58)			

Abbreviations: μg, microgram; aHR, adjusted hazard ratio; FCP, fecal calprotectin; g, gram; IV, intravenous; UC, ulcerative colitis.

a Analysis performed in patients with available data on FCP.

**Table 3:** Sensitivity analysis with outcome ascertainment after 8 weeks in patients with UC on Upadacitinib low dose (15 mg) versus high dose (30 mg) for maintenance treatment after propensity score matching.

Outcomes	Cohorts	*N* (%)	aHR	95% CI	*P* value
**Composite outcome**	Low dose	58 (20.7)	1.36	0.91-2.02	.12
	High dose	42 (15)			
**IV steroid use**	Low dose	57 (20.3)	1.37	0.91-2.04	.78
	High dose	41 (14.6)			
**Colectomy**	Low dose	<10	—	—	—
	High dose	<10			
**Oral steroid use**	Low dose	86 (30.7)	1.12	0.78-1.62	.51
	High dose	79 (28.2)			
**Change in therapy**	Low dose	45 (16)	0.88	0.56-1.37	.56
	High dose	50 (17.8)			
**FCP < 250 μg/g** ^a^	Low dose	40 (56.3)	—	—	.29
	High dose	58 (64.4)			
**C reactive protein < 8 mg/L** ^a^	Low dose	59 (51.7)	—	—	.34
	High dose	70 (57.8)			

Abbreviations: μg, microgram; aHR, adjusted hazard ratio; FCP, fecal calprotectin; g, gram; IV, intravenous; UC, ulcerative colitis.

a Analysis performed in patients with available data on FCP and CRP.

**Table 4:** Comparison of outcomes in patients with UC on Upadacitinib low dose (15 mg) versus high dose (30 mg) for maintenance treatment in patients exposed to two or more advanced therapies after propensity score matching.

Outcomes	Cohorts	*N* (%)	aHR	95% CI	*P* value
**Composite outcome**	Low dose	56 (42.4)	1.03	0.71-1.50	.86
	High dose	54 (40.9)			
**IV steroid use**	Low dose	29 (21.9)	1.19	0.69-2.03	.52
	High dose	25 (18.9)			
**Colectomy**	Low dose	<10	—	—	—
	High dose	<10			
**Oral steroid use**	Low dose	44 (33.3)	0.97	0.64-1.48	.91
	High dose	44 (33.3)			
**Change in therapy**	Low dose	26 (19.6)	1.31	0.73-2.36	.35
	High dose	20 (15.1)			
**FCP <250 μg/g** ^a^	Low dose	18 (50)	—	—	.80
	High dose	18 (52.9)			
**C reactive protein <8 mg/L** ^a^	Low dose	30 (50.8)	—	—	.34
	High dose	34 (59.6)			

Abbreviations: μg, microgram; aHR, adjusted hazard ratio; FCP, fecal calprotectin; g, gram; IV, intravenous; UC, ulcerative colitis.

a Analysis performed in patients with available data on FCP and CRP.

### Predictors of failure of low dose maintenance upadacitinib

**Figure 2:**
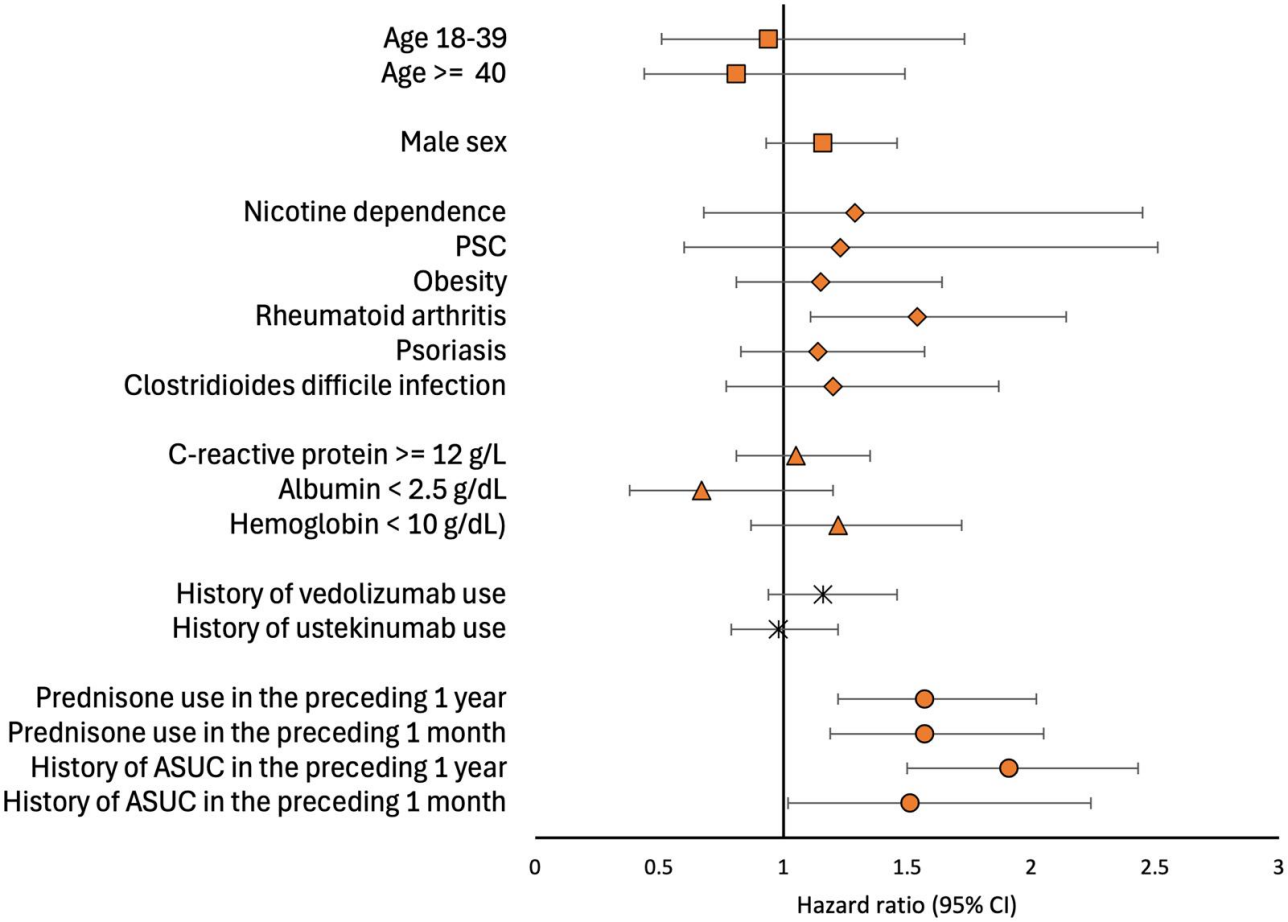
Forest plot of hazard ratios with 95% confidence intervals of predictors of failure in the low dose cohort.

In the Cox proportional hazard model, patients with prednisone use in the preceding 1 year (aHR 1.57, 95% CI, 1.22-2.02) and preceding 1 month (HR 1.57, 95% CI, 1.19-2.05) predicted failure in the low dose cohort. Patients with intravenous steroid use in the preceding 1 year (aHR 1.91, 95% CI, 1.50-2.43) and preceding 1 month (HR 1.51, 95% CI, 1.02-2.24) also predicted failure in the low dose cohort. History of rheumatoid arthritis also predicted failure in the low dose cohort (HR 1.54, 95% CI, 1.11-2.14). The same co-variates were also associated with failure in the high dose cohort (Table 5). Age, sex, nicotine dependence, PSC, obesity, CRP ≥ 12 g/L, Hb < 10 g/dL, albumin < 2.5 g/dL, and Clostridioides difficile infection did not predict failure in low dose or high dose cohort (Table 5; Figure 2).

**Table 5:** Predictors of failure in patients with UC on 15 mg and 30 mg Upadacitinib for maintenance therapy.

Co-variates	Upadacitinib 15 mg			Upadacitinib 30 mg		
	HR	95% CI	Coefficient	HR	95% CI	Coefficient
**Age 18**-**39**	0.94	0.51-1.73	−0.05	1.03	0.59-1.80	0.03
**Age ≥40**	0.81	0.44-1.49	−0.20	0.79	0.45-1.37	−0.23
**Male sex**	1.16	0.93-1.46	0.15	1.13	0.92-1.39	0.12
**Nicotine dependence**	1.29	0.68-2.45	0.25	1.14	0.81-1.62	0.13
**PSC**	1.23	0.60-2.51	0.21	1.08	0.65-1.81	0.08
**Obesity**	1.15	0.81-1.64	0.14	1.20	0.93-1.55	0.18
**CRP ≥12 g/L**	1.05	0.81-1.35	0.04	0.95	0.76-1.18	−0.04
**Albumin <2.5 g/dL**	0.67	0.38-1.20	−0.38	1.27	0.92-1.76	0.24
**Moderate-severe anemia (Hb <10 g/dL)**	1.22	0.87-1.72	0.20	1.17	0.92-1.49	0.15
**Clostridioides difficile infection**	1.20	0.77-1.87	0.18	0.86	0.64-1.16	−0.14
**History of vedolizumab use**	1.16	0.93-1.46	0.15	1.20	0.96-1.51	0.18
**History of ustekinumab use**	0.98	0.79-1.22	−0.015	0.88	0.71-1.10	−0.11
**Concomitant rheumatoid arthritis**	**1.54**	**1.11**-**2.14**	0.43	**1.53**	**1.10**-**2.14**	**0.43**
**Concomitant psoriasis and/or psoriatic arthritis**	1.14	0.83-1.57	0.13	1.08	0.79-1.49	0.08
**Prednisone use in the preceding 1 year**	**1.57**	**1.22**-**2.02**	0.45	**1.56**	**1.24**-**1.96**	**0.44**
**Prednisone use in the preceding 1 month**	**1.57**	**1.19**-**2.05**	0.45	**1.68**	**1.35**-**2.09**	**0.52**
**History of ASUC in the preceding 1 year**	**1.91**	**1.50**-**2.43**	0.64	**1.53**	**1.22**-**1.92**	**0.42**
**History of ASUC in the preceding 1 month**	**1.51**	**1.02**-**2.24**	0.41	**1.58**	**1.17**-**2.15**	**0.46**

Abbreviations: ASUC, acute severe ulcerative colitis; CRP, C-reactive protein; dL, deciliter; g, gram; HR, hazard ratio; L, liter; PSC, primary sclerosing cholangitis; UC, ulcerative colitis.

### Adverse events

Fewer than 10 patients in the low dose and high dose cohorts developed herpes zoster, venous thromboembolism, and major adverse cardiovascular events. There was no difference in the proportion of patients who developed de-novo acne in the low dose versus high dose cohort (17 [3.2%] for 15 mg dosing vs 33 [2.9%], *P* = .74).

## Discussion

Upadacitinib is an effective therapy for the management of ulcerative colitis. In this retrospective cohort study using a multi-institutional database, we assessed the impact of high dose versus low dose upadacitinib on the risk of a composite outcome including hospitalization requiring intravenous steroids, oral steroid use, and/or colectomy between 12 and 60 weeks following the index upadacitinib prescription. Our results indicate no significant difference in the composite outcome of intravenous steroids, oral steroids or colectomy between the high dose and low dose cohorts. We also found no difference in the risk of IV steroid use, oral steroid use, and change in therapy between the two cohorts. We did, however, find that the use of steroids, either oral or IV, in the preceding one year and preceding 1 month prior to upadacitinib predicted failure in the both the low dose and high dose cohort. Additionally, a history of rheumatoid arthritis was also associated with treatment failure in both the cohorts.

Real world data comparing low versus high dose upadacitinib for UC maintenance therapy is limited. Clinical efficacy and safety data comparing low versus high dose upadacitinib comes from the initial maintenance clinical trial.^2^ The primary endpoint of clinical remission at week 52 was achieved by 40.4% in the upadacitinib low dose versus 53.6% for the upadacitinib high dose (*P* < .001 for both compared to placebo).^2^ With regard to adverse events, herpes zoster rates were 6.0/100 person-years and 7.3/100 person-years in the low and high dose upadacitinib, respectively (vs none in the placebo). Malignancy rates, excluding non-melanoma skin cancer, were 0.5/100 person-years and 0.9/100 person-years in low dose and high dose upadacitinib respectively compared to 0.7/100 person-years in placebo. Non-melanoma skin cancer rates were 1.4/100 person-years in high dose upadacitinib with no cases in the low dose upadacitinib group or placebo.^7^ In our study, we evaluated the risk of herpes zoster, malignancy, venous thromboembolism, and major adverse cardiovascular events between the two cohorts. However, given low event rates and obfuscation of patient counts less than 10, we were not able to report the data. There is, however, a retrospective real-world study conducted on tofacitinib, a pan-JAK inhibitor, evaluating 162 patients with ulcerative colitis, of which 52% continued on the high dose regimen of 10 mg twice daily, while 48% underwent de-escalation to 5 mg twice daily. In the dose de-escalation group, 56% patients had a UC disease activity-related event (including hospitalization, surgery, steroid initiation, or tofacitinib dose increase) within 12 months.^9^ This study shows that in contrast to upadacitinib, lower dose of tofacitinib is associated with increased disease activity. This study is, however, a de-escalation study and therefore did not examine predictors of failure as was done in the present study.

Our study has several limitations. First, because of the nature of the database, granular clinical data such as stool frequency and rectal bleeding are not available. In addition, claims data, hospitalization records, and data from external providers data are not available. Furthermore, we are unable to evaluate the endoscopic response to upadacitinib low dose versus high dose. We did, however, measure the proportion of patients who had fecal calprotectin < 250 μg/g in patients with available data, which has been shown to be reliable marker of endoscopic healing. Our outcome analysis was also limited to a 15-month follow-up. Hence, further studies are needed to evaluate more long-term outcomes of upadacitinib low versus high dose dosing. Furthermore, our study is limited as we did not specify that each patient was started on a dose of upadacitinib 45 mg and data on extended induction periods was not available. However, after PSM, there were approximately 10% of patients who had a co-morbid diagnosis of rheumatoid arthritis, psoriasis and ankylosing spondylitis. If the patient was started on upadacitinib for another indication, it would only be for a small proportion of the population. Finally, as with all database studies, there is always a concern for misdiagnosis, residual confounding and under-reporting of some variables. Despite the present limitations, our study also has important strengths. It is one of the few studies to examine the difference in outcomes with regards to differing doses of upadacitinib maintenance therapy. It is also one of the few studies evaluating potential risk factors for failure of low and high dose upadacitinib. Additionally, we had a diverse cohort from HCOs (academic and non-academic) across the United States allowing for generalizability when interpreting the results. We utilized propensity score matching to reduce the impact of confounding variables despite the retrospective nature of the study.

## Conclusion

In summary our study indicates that 15 mg upadacitinib shows similar efficacy to 30 mg upadacitinib, for maintenance treatment of UC with regard to our compositive outcome of intravenous steroids, oral steroid use and/or colectomy, using a real-world database. However, recent oral or IV steroid use as well as comorbid rheumatoid arthritis predicted failure of both 15 mg and 30 mg upadacitinib cohorts. Further studies are needed to understand long term efficacy and impact on endoscopic disease activity.

## Data Availability

The data underlying this article will be shared on reasonable request to the corresponding author.
